# Advancing Knowledge of Values-Clarification Processes During Complex Decision-Making Among Older Adults With Advanced Cancer: Protocol for a Pilot Randomized Trial Using Simulated Patient-Clinician Encounters

**DOI:** 10.2196/80531

**Published:** 2025-10-27

**Authors:** Amy C Cole, Allison M Deal, Angela M Stover, Lisa Vizer, Fei Yu, Andy J King, Lukasz Mazur, Daniel R Richardson

**Affiliations:** 1 Division of Healthcare Engineering, Department of Radiation Oncology School of Medicine University of North Carolina at Chapel Hill Chapel Hill, NC United States; 2 Carolina Health Informatics Program University of North Carolina at Chapel Hill Chapel Hill, NC United States; 3 UNC Lineberger Comprehensive Cancer Center Chapel Hill, NC United States; 4 Gillings School of Global Public Health University of North Carolina at Chapel Hill Chapel Hill, NC United States; 5 Huntsman Cancer Institute University of Utah Salt Lake City, UT United States

**Keywords:** human factors, oncology, values-clarification, shared decision-making, communication, simulations

## Abstract

**Background:**

Older adults represent the majority of individuals diagnosed with cancer in the United States and often face complex treatment decisions that require balancing survival benefits with quality-of-life considerations. Despite the emphasis on shared decision-making (SDM), many patients report that clinical guidance does not reflect their personal values. Values-clarification tools have shown slight improvements in aligning care with patient values; however, the factors by which these tools influence decision-making are less studied.

**Objective:**

This pilot study aims to estimate the quality of values-clarification and SDM processes that occur during simulated diagnosis encounters for advanced cancer among participants who do and do not receive a values-clarification tool.

**Methods:**

Pilot randomized study using simulated patient-clinician encounters to assess how values are elicited, processed, and integrated into treatment decisions. Participants aged 60 years and older with an advanced cancer diagnosis will be randomized to receive either a digital values-clarification instrument codeveloped through stakeholder engagement, referred to as Values and Outcomes to Improve Cancer Experiences (VOICE), or a general communication guide from the American Cancer Society (ACS). Each participant will engage in 2 simulated encounters (values-based and nonvalues-based), conducted by trained medical students portraying oncologists. The simulations are structured using a situational awareness framework. A novel rating scale, referred to as VECTORS (Values Elicitation and Clarification of Treatment Options Rating Scale), will measure the quality of rapport building, clinician engagement, and patient engagement through observation. Additional validated instruments will be used to quantify observed (OPTION-5) and patient-reported (CollaboRATE) SDM behaviors and patients’ perceived usefulness of either VOICE or the ACS guide (PrepDM [Preparation for Decision-Making]). Qualitative interviews will be used to better understand participants’ experiences during encounters and perceptions of VOICE or the ACS guide.

**Results:**

The study was conducted between September 2024 and December 2024, with a total of 44 participants, and is ready for data analysis.

**Conclusions:**

This pilot study will provide preliminary evidence into the quality of values-clarification processes occurring during patient-clinician encounters among older adults making treatment decisions for advanced cancer.

**International Registered Report Identifier (IRRID):**

DERR1-10.2196/80531

## Introduction

In 2025, over 2 million people in the United States are expected to be diagnosed with cancer [1], with older adults (aged ≥60 years) comprising the majority of these diagnoses [2]. While cancer is the leading cause of death for older adults [1], their survival rates are improving, making up 78% of the more than 18 million people living with cancer [3]. These advances are positive, but also introduce greater complexity into decision-making [4], as older adults must weigh the benefits of survival to the risks to their quality of life [5]. For those with advanced cancers [6], particularly older adults with comorbidities, treatment pathways can vary and are often influenced by personal values [6,7].

Many older adults report that the guidance they receive from their clinicians does not align with their values when making a treatment decision [8-14]. Patient “values” are the unique preferences, concerns, and expectations a patient brings to a clinical encounter, which should be integrated into clinical decisions for effective patient care [12]. When patients do not engage in values-based communication with their clinicians, it can lead to decisions that negatively impact their quality of life [15], including undergoing aggressive treatments due to patients’ unrealistic expectations of a cure [12,16,17].

Shared decision-making (SDM) guidelines advocate for aligning treatment plans with patient values [14,18-22], and values-clarification tools have shown slight improvements in values-discordance [20]. Research has often focused on decision-making outcomes rather than the process itself, leaving the values-clarification processes that occur during clinical encounters understudied [23,24]. Therefore, to advance knowledge of how patients’ values are discussed, understood, and incorporated into the treatment decision, we applied a situational awareness framework. Situational awareness characterizes an individual’s ability to gather information, comprehend information, and anticipate what will occur after making a decision based on that information [25]. Situational awareness can be assessed using simulations and has been used in health care to identify communication gaps and training opportunities [26-29]. Therefore, applying the situational awareness framework to values-clarification and SDM provides a structured approach to evaluate engagement quality, highlight missed communication opportunities, and conceptualize values-discordance as a potential safety risk given its link to higher treatment burden [21] and more aggressive care [18,22].

This protocol describes a pilot study to estimate the quality of values-clarification and SDM processes during simulated diagnosis encounters for older adults (aged ≥60 years) with advanced cancer, among participants who do and do not receive a values-clarification tool. Using a situational awareness framework, we aim to evaluate how these processes unfold, where they can be strengthened, and how a values-clarification tool may influence engagement. These aims guide the following primary research questions: (1) Do participants in the intervention group experience higher quality values-clarification processes compared to the control group, as measured by a standardized rating scale? (2) Does participant use of the intervention tool influence clinician engagement in values-clarification processes, as measured by a standardized rating scale? (3) Are participants in the intervention group more likely than the control group to initiate discussions about their values during simulated encounters? (4) Does participant initiation of values (compared to clinician initiation) have an impact on the quality of values-clarification processes, as measured by a standardized rating scale?

These aims also guide the following secondary research questions: (1) Does the intervention improve both observed and patient-reported SDM experiences and perceived preparedness for decision-making, compared to the control group? (2) What themes emerge from qualitative data regarding patients’ experiences of value-clarification and decision-making processes during simulated encounters?

## Methods

### Prior Work

We previously developed the Values and Outcomes to Improve Cancer Experiences (VOICE) tool, a digital values-clarification instrument intended to support SDM in oncology settings. To inform the development of VOICE, we conducted a stakeholder-driven concept mapping study involving 7 older adults with advanced cancer, 2 caregivers, and 12 oncology specialists [30,31]. VOICE uses best-worst scaling (BWS) to elicit and prioritize treatment values (ie, what patients value most when making treatment decisions). VOICE also generates a tailored summary report that includes question prompt lists aligned with each patient’s top-ranked treatment values [30,31]. The tailored summary report was designed to prompt patients to discuss their values, ask questions, and express in their own words how the treatment options may affect what matters most to them.

### Study Design

**Figure 1:**
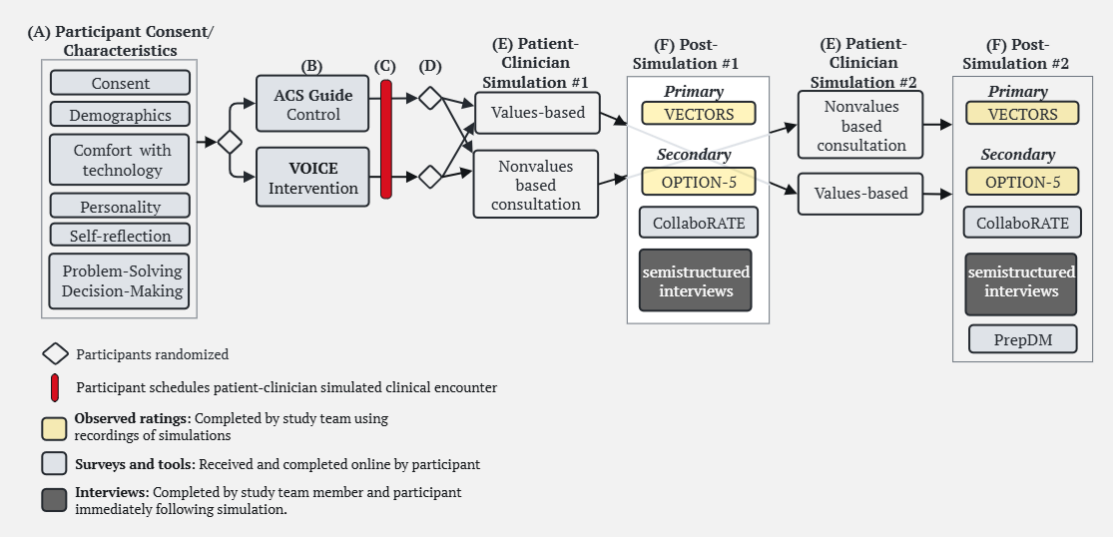
Randomized pilot testing study schema. (A) Participants will complete informed consent in REDCap (Research Electronic Data Capture) and participant characteristics in Qualtrics remotely before scheduling to engage in simulations. (B) To ensure balanced participation across the 4 potential pathways, a systematic alternating method in Qualtrics will be used to assign participants to the intervention (VOICE) or control (ACS) groups. (C) After participants complete and review VOICE or the ACS guide, they are prompted to contact the research team to schedule the patient-clinician simulated encounters. Patients are reminded they are preparing for a simulated patient-clinician encounter in which they will be asked to discuss and make a decision regarding a “mock” secondary cancer diagnosis. (D) The order of encounter type (VB vs NVB) will be random using a random number generator in Microsoft Excel. (E) All participants will engage in two (20- to 30-minute) simulated encounters (VB and NVB) in random order, both occurring on the same day. Clinicians (ie, medical students portraying oncologists) will only be blinded as to whether the participants received VOICE or the ACS guide prior to the interaction. Clinicians will be informed of the encounter type to ensure they follow appropriate scripts. Simulated encounters will be held at a single site in the Simulation, Experiential Learning, and Training (SET) Center located at the University of North Carolina at Chapel Hill School of Medicine. Simulated patient-clinician encounters will be audio-video recorded. (F) Participants will complete surveys (CollaboRATE and PrepDM [Preparation for Decision-Making]) and engage in semistructured interviews immediately following each simulation. Our research team will complete observed ratings of encounters (VECTORS and OPTION-5) after simulations have concluded by reviewing the audio-recorded simulations. ACS: American Cancer Society; VECTORS: Values Elicitation and Clarification of Treatment Options Rating Scale; VOICE: Values and Outcomes to Improve Cancer Experiences.

We will conduct a double-blinded randomized pilot study using 2 types of simulated patient-clinician encounters (values-based [VB] and nonvalues-based [NVB]) between an intervention group that will receive VOICE and a control group that will receive an American Cancer Society (ACS) communication guide. Participants randomized to VOICE will complete a series of 7 BWS questions, then receive a tailored summary report based on their values, within Qualtrics. Completing VOICE typically takes up to 8 minutes. See Multimedia Appendix 1 for an example of the BWS instrument and tailored summary report. For this study, we selected the ACS guide as the control condition. Participants randomized to the control group will receive the ACS guide in Qualtrics and typically takes up to 5 minutes to review. The ACS guide was developed as a tool to improve patient-clinician communication and includes a general list of cancer-related question prompts. By comparing VOICE to the ACS guide, we aim to isolate whether tailored summary reports that are aligned with patient values better prepare patients to engage in values-based discussions with their clinicians. Therefore, if VOICE effectively prepares patients, it may prompt clinicians to engage in values-based discussions, subsequently influencing clinician behavior. This conditional structure is embedded in the nonvalues-based simulation script design (see the Simulation Scripts section). The study design schema is described and depicted in Figure 1.

### Study Participants

We will identify potential participants by reviewing inpatient census and clinic schedules within Epic and contacting them by phone and email. Participants' recruitment eligibility includes (1) confirmed cancer diagnosis at an advanced stage, not limited by cancer site, (2) being able to read and understand English, (3) being age ≥60 years, and (4) being willing to provide consent. Participants with dementia, altered mental status, or psychiatric conditions that would prohibit the understanding of informed consent or participation in a simulated encounter are not eligible to enroll in the study. With a study team member, participants will review and complete informed consent in person or by phone before enrollment and review it again upon arrival for the simulations.

### Ethical Considerations

This study was reviewed and approved by the University of North Carolina at Chapel Hill (approval number 24-1566). Participant consent will be obtained electronically through REDCap (Research Electronic Data Capture; Vanderbilt University). Participants will receive US $100 for their participation. Survey data will be recorded in REDCap, a secure web application. Survey data, as well as recordings from mock consultations and interviews, will be deidentified and stored on a secure HIPAA (Health Insurance Portability and Accountability Act)-compliant location managed by the University of North Carolina School of Medicine. Protections will be taken to ensure that confidentiality is maintained, ensuring that all personnel are trained on HIPAA regulations, and restricting data access to study personnel.

### Simulation Preparation

To conduct the simulated encounters, we (1) developed disease briefs, (2) trained medical students, (3) developed 2 simulation scripts, and (4) used a modified situational awareness rating scale to estimate the quality of values-clarification processes.

#### Disease Briefs

Textbox 1 describes 2 disease briefs developed and reviewed by 3 clinical oncologists. Disease briefs included a mock cancer diagnosis and treatment options (delivery, side effects, and efficacy).

**Disease briefs for simulated patient-clinician encounters.**

**Disease brief 1**

*Cancer diagnosis: “Leukocythemia”—aggressive blood cancer*

- **Treatment option 1 (standard option):** Combination chemotherapy
  Delivery: Infusions 7 days in a row at the infusion center every month plus a chemo pill
  Side effects: Fatigue, risk of hospitalization, weakness; 95% of patients are admitted for complication of treatment at some point in time
  Efficacy: Median survival of 12 months
- **Treatment option 2 (medically inferior option):** Oral chemotherapy
  Delivery: Oral pill taken every day
  Side effects: None
  Efficacy: Median survival of 8 months

**Disease brief 2**

*Cancer diagnosis: “Encephaloid tumor”—lung cancer*

- **Treatment option 1 (standard option):** IV chemotherapy
  Delivery: IV chemo weekly
  Side effects: Low blood counts, infection risk, weakness (100% of patients)
  Efficacy: Median survival of 4-5 years
- **Treatment option 2 (medically inferior option):** Oral chemotherapy
  Delivery: Oral chemo every day
  Side effects: Constipation (50%), muscle pain (50%), joint pain (50%), weakness (100%)
  Efficacy: Median survival of 4-5 years

#### Medical Student Training

Five students from the University of North Carolina School of Medicine were recruited and trained to portray oncologists in the simulated patient-clinician encounters. A member of the research team served as a standardized patient during training. The training process included 2 hours of simulation script review with our research team, 1 hour of observation of an oncologist conducting both VB and NVB simulated encounters, and 6 hours of in-person practice, during which students practiced both types of simulations. To ensure consistency throughout the study, students will be debriefed after each simulated encounter to reinforce standardized engagement with participants.

#### Simulation Scripts

We developed 2 simulation scripts (values-based and nonvalues-based), with guidance from a simulation expert and 3 clinical oncologists.

#### Values-Based Simulation Script

A review of literature was performed to assess the best practices in communication and SDM during clinical encounters [21,32-37]. The resulting VB script was designed to model a “high-quality” encounter in which clinicians focus on discussing and incorporating patients’ values into treatment decisions. The quality of the encounter was structured using three levels of situational awareness [38], modified for clinician and patient engagement.

- **Level 1—Gathering Information:** Identifying what matters most to the patient when making a treatment decision.
- **Level 2—Processing Information:** Exploring how the patient’s values are impacted by treatment options.
- **Level 3—Projecting Information:** Anticipating what will occur after making a decision based on that information.

This script was developed to ensure the oncologist addressed all 3 levels during the encounter, supporting a values-aligned decision-making process.

#### Nonvalues-Based Simulation Script

The NVB simulation script was designed to model a “lower quality” encounter in which the oncologist focuses on treatment decisions without explicitly addressing the patient’s values. In these encounters, any shift toward values-based discussion must be initiated by the patient, referred to as “patient agency.”

For example, if a participant mentions caring for their grandkids and expresses concern about how treatment may affect that role, the clinician is prompted to transition to level 2 of the VB framework (processing information about how the patient’s values are impacted). This may also prompt the clinician to go to level 1 (gathering information about what the patient values most). After addressing the patient’s concern, the clinician returns to the NVB script to inquire if the patient is then ready to make a treatment decision. During NVB encounters, clinicians are instructed not to advance through the values-based levels unless explicitly prompted by the patient. This structure ensures that values-based engagement in NVB encounters occurs only in response to patient-initiated cues.

**Figure 2:**
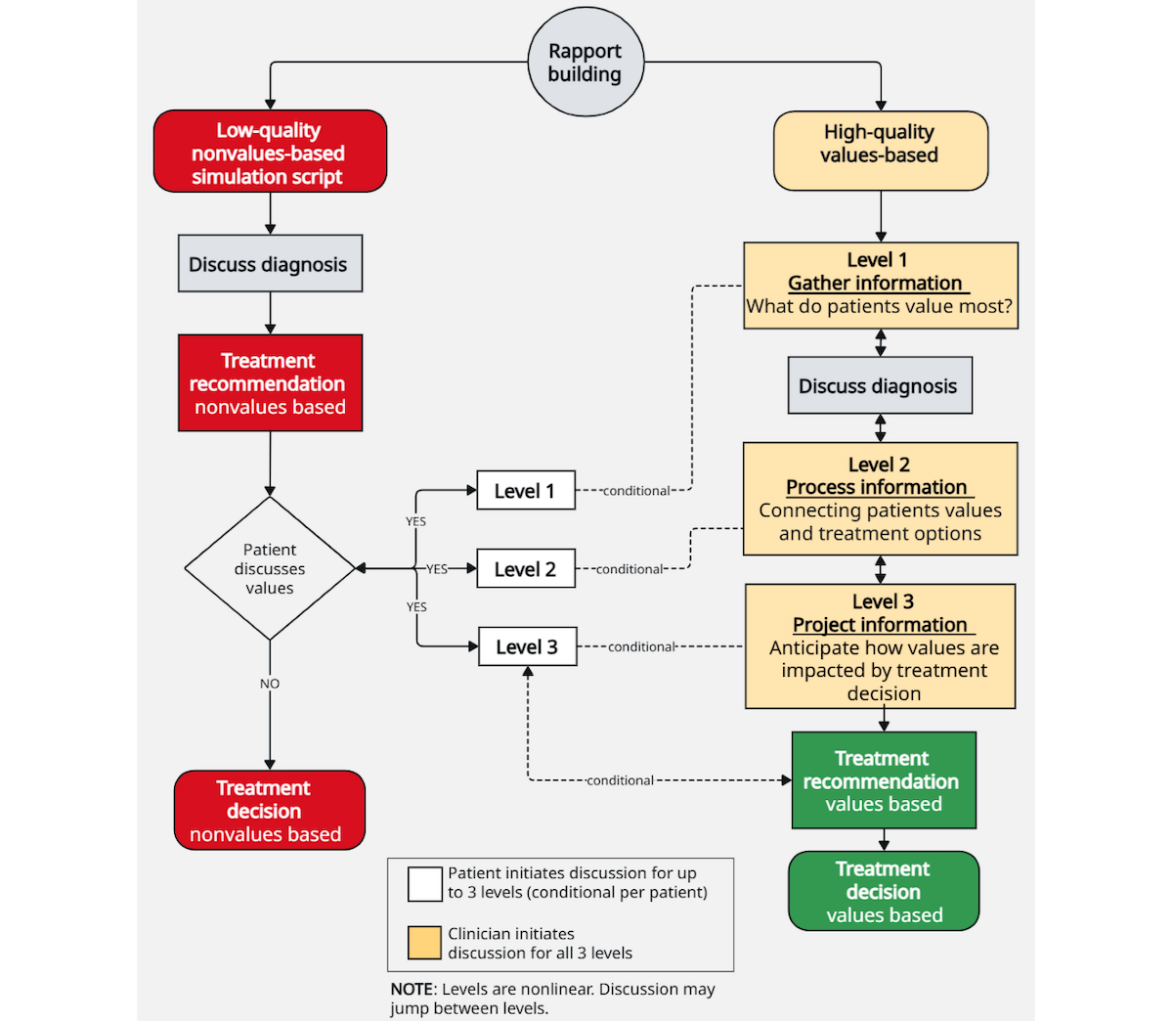
Simulation script schema.

See Figure 2 for the simulation script schema. This schema was used to support both the development of simulation scripts and medical student training for conducting VB and NVB encounters.

#### Values Elicitation and Clarification of Treatment Options Rating Scale

We developed a novel rating scale called the Values Elicitation and Clarification of Treatment Options Rating Scale (VECTORS). VECTORS is a 7-item rating scale that includes 3 domains: rapport building (1 item), clinician engagement (3 items), and patient engagement (3 items). VECTORS uses a Likert-type scale with 3 response options representing a 10-point scale (0=no effort observed, 5=moderate effort was observed, and 10=exemplary effort was observed). Higher scores reflect higher quality of values-clarification processes.

Table 1 displays the rating score criteria for each of the 7 items in VECTORS. VECTORS was established following the same situational awareness framework and review of literature as described under the simulation script development to rate the quality of values-clarification processes that occurred during patient-clinician encounters.

**Table 1:** Values Elicitation and Clarification of Treatment Options Rating Scale (VECTORS).

Rating score			Scoring criteria	Rating example
**Rapport building (clinicians and patients engaged in rapport building)**				
	0		Patients and clinicians did not engage in rapport building.	N/A^a^
	5		Patients and clinicians engaged in rapport building moderately well, but only surface-level discussion.	*Clinician*: “*How are you doing today?, How was your drive in?, etc.”Patient*: “*I’m doing okay, it’s a nice day outside, and the drive in wasn’t too bad.”*
	10		Patients and clinicians engaged in rapport building extremely well. Patients and clinicians engage in a dialogue to learn more about one another.	*Clinician*: *“Tell me about yourself, what are some of things that you enjoy doing*.” *Patient*: “*I’m 62 years old, married and have 2 kids. I enjoy going for walks with my dog, playing the violin and going out to restaurants with my friends*.” *Clinician*: “*That sounds very enjoyable. I appreciate you sharing that with me. I hope to learn more about you as we go through this journey together. I’d like to tell you more about myself, and the role that I will have in your cancer care.”*
**Clinician engagement**				
	**Level 1: Gather information (clinicians discussed what their patients value most)**			
		0	Patients’ values not discussed, no reference or acknowledgment.	N/A
		5	Discussed moderately well. Clinician initiates discussion to understand how the patients’ values might inform a treatment decision but does not follow up with questions to learn more about what that means to the patient. Additional conversation does not ensue to understand how their values would be applied to making a treatment decision.	*Clinician**:** “I would like to talk about what you value most when thinking about treatment options as this can help us decide on the treatment pathway that is best for you. Patient: [responds]. Clinician: “Thank you for letting me know, that’s important for us to think about when making a treatment decision.”*
		10	Discussed extremely well. Clinician initiates a discussion to understand how their patients’ values might inform a treatment decision. Follows up with questions to learn more about what that means to the patient.	*Clinician:* “*I would like to talk about what you value most when thinking about treatment options as this can help us decide on the treatment pathway that is best for you. Would that be okay if we talked about that?” Patient: [responds]. Clinician: “Can you tell me what maintaining your day to day activities mean to you. What concerns do you have about this?”*
	**Level 2: Process information (clinicians discussed how treatment options may impact what patients value most)**			
		0	Not discussed, presented treatment options, but did not mention how the patients’ values may be impacted.	N/A
		5	Discussed moderately well.	Treatment options were presented, but the conversation only addressed how the clinician’s recommended option might affect what the patient values.
		10	Discussed extremely well.	Treatment options are presented, and the conversation addressed how each option might affect what the patient values.
	**Level 3: Project information (clinicians confirmed their patient understood how treatment options may impact what they value most)**			
		0	Not discussed, no reference or acknowledgment.	N/A
		5	Discussed moderately well. Clinician asks a simple question confirming understanding of the treatment the clinician recommended.	*Clinician:* “*Do you have questions about this treatment?”*
		10	Discussed extremely well. Clinician confirms the patient understood or helps clarify any misunderstandings.	*Clinician:* “How would you describe both treatment options to your family?”, or “In your own words, how will each treatment impact the values we previously discussed?” or “In your own words, what does choosing this treatment mean to you?”
**Patient engagement**				
	**Level 1: Gather information (patients discussed what they value most)**			
		0	Patients’ values not discussed, no reference or acknowledgment.	N/A
		5	Discussed moderately well. Patient initiates discussion to discuss what they value most. Patient does not explain why this is important to them. Additional conversation does not ensue to clarify why this is important to them.	*Patient: “Maintaining my day-to-day activities is important to me.” Clinician: [responds]. Note: Clinicians’ response varies based on their engagement level.*
		10	Discussed extremely well. Patient initiates discussion to discuss what they value most. Patient provides examples of why this is important to them. Additional conversation ensues to clarify why this is important to them.	*Patient:* “*Maintaining my day-to-day activities is important to me because I want to...” Clinician: [responds]. Note: Clinicians’ response varies based on their engagement level.*
	**Level 2: Process information (patients discussed how treatment options may impact what they value most or asks questions to clarify how their values could be integrated into treatment decisions)**			
		0	Not discussed, no reference or acknowledgment.	N/A
		5	Discussed moderately well. Patient discusses or asks questions about how *what they value* may be impacted by treatment options.	*Patient:“I have some questions about how this will...” Clinician: [responds].***Note:** Patient asked questions but the responses and follow-up conversation do not help to clarify how their values can be applied to making a treatment decision. Patient does not ask additional clarifying questions or engage in additional dialogue.
		10	Discussed extremely well. Patient discusses or asks questions about how *what they value* may be impacted by both treatment options.	*Patient:“I have some questions about how this will...” Clinician: [responds].***Note:** Patient asked clarifying questions or engaged in additional dialogue to ensure the clinician explains how their values can be applied to making a treatment decision.
	**Level 3: Project information (patients confirmed they understood how treatments may impact what they value most)**			
		0	Not discussed, no reference or acknowledgment.	N/A
		5	Moderately well. Patient provides a simple confirmation, either in response to the clinician or by initiating the confirmation themselves.	*Patient:* “*Yes, I understand how each treatment option may impact what I value most*.”
		10	Extremely well. Patient provides examples of how each treatment option may impact what they value most, either in response to the clinician or initiated confirmation themselves.	*Patient:* “*I understand that if I choose treatment x, I may have to rely on a caregiver to help me get dressed, take a bath, etc*.”

### Data Collection

All data will be collected electronically, deidentified, and stored on a secure HIPAA-compliant drive. Only study team members will have access to these data to maintain confidentiality of study participants. Missing or incomplete data will not be imputed or otherwise adjusted; analyses will be conducted using available data only.

#### Participant Characteristics

Characteristic information will include participants’ age, sex, race, ethnicity, cancer diagnosis, education level, household income, employment status, comfort level with technology, self-reflection and insight, and preferred role in problem-solving and decision-making (PSDM).

Self-reflection and insight will be assessed subjectively using the validated Self-Reflection and Insight Scale (SRIS) [39]. The SRIS is a 12-item assessment that measures participants' (1) engagement in self-reflection, (2) need for self-reflection, and (3) insight into self-reflection using a 5-point Likert-type scale. Higher scores indicate higher self-reflection and insight. The SRIS is an appropriate measure to assess participants' perceptions on whether the information in either the VOICE report or the ACS guide was useful for treatment decision-making.

To determine participants’ preferred role in PSDM, mean scores will be calculated for both problem-solving (PS) and decision-making measures for each participant, then placed into 1 of 3 classifications: hand over (mean score <3), share (mean score between 3 and 3.99), or keep (mean score ≥ 4) [40]. Participants who “hand over” both PS and decision-making are categorized as “passive.” Patients categorized as “shared,” either “hand over” or “share” PS, but “share” or “keep” decision-making. Four subcategories exist for patients categorized as “shared”: (1) leaning passive, (2) shared equally, (3) leaning autonomous, and (4) divide and share. Patients categorized as “autonomous” want to retain control of both PS and decision-making (ie, PS equals “keep,” and decision-making equals “share” or “keep”). Two subcategories exist for patients categorized as “autonomous”: (1) leaning shared and (2) consumerist. It is considered theoretically implausible for an individual to assume control for PS, but not for decision-making. Categorization of PSMD roles is displayed in Table 2.

**Table 2:** Participant categorization of problem-solving and decision-making (PSDM) roles.

Decision-making	Problem-solving (PS)		
	Hand over (mean <3)	Share (between 3 and 3.99)	Keep (mean ≥3)
Hand over (mean <3)	Passive	Theoretically implausible	Theoretically implausible
Share (between 3 and 3.99)	Shared (leaning passive)	Shared (equally)	Autonomous (leaning shared)
Keep (mean ≥3)	Shared (divide and share)	Shared (leaning autonomous)	Autonomous (consumerist)

#### Primary Measure—Quantitative

The primary outcome is the quality of values-clarification processes during simulated treatment decision encounters for advanced cancer, assessed using VECTORS to compare intervention (VOICE) and control (ACS) groups, as well as VB and NVB encounters.

Two independent raters will review the recorded simulations and use VECTORS to estimate the quality of values-clarification observed during each encounter. Rater 1 will assist with recordings; therefore, it will not be blinded. Rater 2 will be blinded, as the recorded simulations will not indicate whether simulations were VB or NVB, or whether participants were in the intervention or control groups. We will calculate the Cohen κ coefficient to evaluate interrater reliability. We will conduct a sensitivity analysis by performing data analysis on the dataset that includes ratings from rater 1, then perform the same data analysis using the dataset that includes ratings from rater 2. We will compare the data analysis results to look for differences.

#### Secondary Measures—Quantitative

Data from secondary measures will either be collected through observed ratings by study team members (VECTORS and OPTION-5), participant surveys (CollaboRATE and PrepDM), or interviews, as described below and depicted in Figure 1.

SDM communication behaviors will be subjectively assessed using the OPTION-5 scale [36,37]. OPTION-5 is a validated scale used to assess the extent to which SDM communication behaviors occur during an initial diagnosis encounter. OPTION-5 is a 5-item validated survey that has been used to measure the extent to which clinicians involve patients in treatment decisions, including oncology patients, and is therefore an appropriate assessment for this study [37,41-44]. Five aspects of the clinicians’ behavior during the simulated encounters will be evaluated and rated using a 5-point Likert scale, ranging from “0=no effort observed” to “4=exemplary effort is observed” [37]. The five aspects include the extent to which the clinician (1) draws attention to or confirms alternate treatment options exist, (2) supports the patient to become informed or deliberate about treatment options, (3) gives information or checks understanding about the options available, (4) elicits the patients’ preferences about the options available, and (5) makes an effort to integrate the patients' preferences into the treatment decision [36,37]. Two independent raters will use the OPTION-5 scale to assess the level of SDM observed during each simulated patient-clinician encounter, as described for VECTORS. Interrater reliability and sensitivity analyses will be performed, as described for VECTORS.

The level of SDM that occurs in the simulated encounters from the patient’s perspective will be subjectively assessed immediately following simulations using the 3-item validated CollaboRATE scale [35]. CollaboRATE is a patient-reported assessment that measures patients’ perceived effort of clinicians to (1) understand patients’ health issues, (2) listen to what matters most about their health issues, and (3) include what matters most when choosing a treatment [35].

Usefulness in preparing participants to engage in SDM will be measured immediately following the final simulation using the PrepDM scale. PrepDM scale [45], a validated 10-item scale, will be used to assess participants’ perceived usefulness of the tool in preparing them to communicate with oncologists during the simulated encounter.

Performance actions of participants will be assessed after encounters, including (1) whether participants brought either the tailored digital summary report from VOICE or the ACS guide to the encounter and (2) whether participants informed the clinician they completed and reviewed VOICE or the ACS guide.

#### Secondary Measures—Qualitative

All participants will engage in 2 semistructured interviews immediately following each simulation to assess (1) situational awareness (ie, ability to gather, process, and project information) [38] during the simulated encounter and (2) perceptions of whether VOICE or the ACS guide prepared them for the encounter. Qualitative data from postsimulation interviews will be summarized and grouped into themes, which will be used to understand the impacts of using VOICE or the ACS guide prior to an encounter.

### Data Analysis

Quantitative analyses will be performed in RStudio, and qualitative analyses will be performed in MAXQDA.

#### Sample Size and Power

This pilot study was not powered, as its primary objective was to gather preliminary data necessary to inform the design and power calculations of a future, larger-scale study. We aimed to estimate the quality of values-clarification processes during simulated encounters (as measured by VECTORS, ranging from 0 to 100) for intervention and control groups in 2 different simulated encounters (VB and NVB). We will attempt to enroll 11 participants in each pathway to gather preliminary data, for a total of 44 participants.

The preliminary data gathered from this pilot study, as well as other study outcome measures, will inform future work using VECTORS in real-world and simulated encounters.

#### Participant Characteristics

Descriptive statistics will be used to summarize participants’ demographic characteristics, comfort level with technology, personality (BFI-2-XS [Big Five Inventory-2 Extra-Short Form]) [46], self-reflection and insight (SRIS) [39,47], and preferred role in PSDM [40].

#### Primary Outcome—Quantitative

Simulated encounters will be quantified using VECTORS to assess the estimated quality of values-clarification processes that occur between intervention and control groups during both VB and NVB encounters. Interrater reliability for VECTORS will be assessed by calculating the Cohen κ weighted coefficient and discrepancies in ratings. Kappa statistics between 0.61 and 0.80 indicate substantial agreement between raters, and 0.81 to 1.00 indicate almost perfect agreement between raters [48]. Analyses of the 3 domains in VECTORS include the raw scores (rapport building: 0-10; clinician engagement: 0-30; patient engagement: 0-30). Analyses of VECTORS overall scores involve taking the sum of all 3 domains, producing a raw score of 0-70, then rescaling to a score of 0-100.

Descriptive statistics will be used to report the mean and SD for VECTORS overall and by each domain. Mean and SD will be reported as cross-tabulations for all encounters, and by intervention and control for VB and NVB encounters, and for whether clinicians transitioned from NVB to VB encounters due to patient agency (ie, patients initiated a discussion pertaining to their values).

Linear regression models using generalized estimating equation methods will be fit using all patient data from both encounter types (VB vs NVB) to account for repeated measures per patient. These models will objectively estimate differences by group and by encounter type and test for potential interactions to show if the intervention works better in one scenario compared to the other.

#### Secondary Outcome—Quantitative

Simulated encounters will be subjectively analyzed using the OPTION-5 instrument to assess the extent to which SDM communication behaviors occurred during the simulation. The OPTION-5 scores from each simulation will be summed, producing a score from 0 to 20, then rescaled to a scale of 0-100. Interrater reliability for OPTION-5 will be assessed as described in the primary outcome for VECTORS.

Patient self-reported scores from the CollaboRATE assessment will be summed, then rescaled to a scale of 0-100. CollaboRATE scores will also be dichotomized (perfect score vs nonperfect score) to allow the use of logistic regression. CollaboRATE often shows ceiling effects; therefore, dichotomizing responses improves the variation among participant scores [49,50].

The PrepDM total mean scores will be quantified by averaging 10 subscales. Items will be summed, then divided by 10 to provide a mean score. Scores will be rescaled to a 0-100 scale by subtracting 1 from the summed score and multiplying by 25, with higher scores representing higher perceived levels of preparation in decision-making [45].

Descriptive statistics will be used to report the mean and SD by intervention and control groups for secondary outcome measures: OPTION-5, CollaboRATE, and PrepDM. Mean and SD will also be reported by VB and NVB encounters for OPTION-5 and CollaboRATE, and by whether participants brought the tool (VOICE or ACS guide) for PrepDM scores.

Descriptive statistics will also be used to summarize the number of participants who (1) brought either the tailored digital summary report from VOICE or the ACS guide to the encounter or (2) informed the clinician they completed and reviewed VOICE or the ACS guide.

Kruskal-Wallis tests will be performed to objectively evaluate differences in distributions in OPTION-5, CollaboRATE, and PrepDM scores.

Linear regression models will be performed for OPTION-5, as described in the primary measure for VECTORS. Log-binomial regression models will be performed with all binary patient data from both types (VB vs NVB) to account for repeated measures per patient. These models will objectively estimate differences by group and by encounter type and test for potential interactions to show if the intervention works better in one scenario compared to the other.

#### Secondary Outcome—Qualitative

Qualitative data from the simulated patient-clinician encounters and postsimulation interviews will be analyzed by 3 members of our research team. The blinded rater responsible for scoring VECTORS and OPTION-5 will not be involved in coding interviews to maintain independence between assessments. The qualitative analysis will follow a hybrid approach with 3 phases [51]. In phase 1, data from interviews will be categorized by a priori themes from the interview guide. In phase 2, a posteriori codes will be created and summaries from participant responses developed, looking for meanings and patterns in the interviews. Discrepancies will be collectively addressed to ensure consensus on codes. In phase 3, the a priori and a posteriori codes will be combined into family codes to structure the findings from the interviews [51]. All codes will undergo a comprehensive review and will be grouped into themes through discussion of the research team.

## Results

The study was conducted between September 2024 and December 2024, with a total of 44 participants, and is ready for data analysis.

## Discussion

### Anticipated Findings

This study addresses a critical gap in understanding how to evaluate the quality of values-clarification processes, specifically how patients’ values are discussed, understood, and incorporated into the treatment decisions. By simulating initial diagnosis encounters and using a situational awareness framework, we will evaluate how these processes unfold and where they can be strengthened, when patients do and do not receive values-clarification tools. This approach will also provide empirical evidence to better understand how a values-clarification tool may influence the quality of values-clarification and SDM processes.

### Strengths and Limitations

This study may be limited by not fully capturing the emotional and contextual complexity of real-world interactions. This study also focuses on short-term outcomes and is unable to provide evidence for the long-term impacts of values-clarification processes on patients’ quality of life or satisfaction with treatment decisions. While there is potential for the Hawthorne effect, where participants alter their behavior due to awareness of being observed, our use of standardized simulations and both observed and patient-reported measures will help mitigate its impact by ensuring consistency and capturing multiple perspectives.

The strengths of this study protocol are its integration of patient-reported measures, observer ratings, and the development of simulations using standardized methodologies. This mixed methods approach provides a more comprehensive understanding, allowing for identification of both alignment and gaps between patients’ expressed values and treatment decisions. Combining evaluations of participant characteristics and secondary measures alongside VECTORS (primary measure) helps provide a more comprehensive understanding of the study participants and will contribute to improvements in values-clarification and SDM processes. Assessing participants’ behaviors and interactions during simulated encounters through VECTORS and a validated assessment (OPTION-5) will lead to a better understanding of what factors may influence participants’ engagement in values-clarification and SDM processes. Personality (BFI-2-XS), self-reflection and insight (SRIS), and preferred role in PSDM measures provide benefit to this research, serving as complementary measures. The SRIS is effective for assessing the differences in an individual’s self-awareness and gives insight into an individual's behavior around decision-making [39]. PSDM serves as a complementary measure to better understand relationships between patients’ perceptions of who should make health care decisions and the actual decisions they make, as observed during the simulated encounters.

By simulating patient-clinician encounters, we control for potential confounding variables, including clinicians’ values-clarification and SDM behaviors, and randomization of information (VOICE vs ACS guide). As current SDM measures alone may not provide evidence on whether patients are actively engaged in SDM [52], simulations of patient-clinician encounters provide the opportunity to evaluate deviations from a “high-quality” SDM encounter.

### Conclusions

This study protocol outlines a structured, simulation-based approach to estimating the quality of values-clarification processes in clinical decision-making. By capturing how patients and clinicians engage in values-clarification processes, this study will provide a replicable framework for studying values-based communication and evaluation of values-clarification tools. Findings from this study may inform clinical practice by identifying communication and intervention strategies that improve how older adults with advanced cancers and clinicians elicit and integrate patients’ values during treatment decision-making. Although this study is conducted in a simulated setting, the framework and insights may be generalizable to clinical contexts and will guide future research and clinical practice aimed at improving SDM.

## Appendix

Multimedia Appendix 1 Example of best-worst scaling (BWS) survey and tailored summary report.

## Abbreviations

ACS: American Cancer Society
BFI-2-XS: Big Five Inventory-2 Extra-Short Form
BWS: best-worst scaling
HIPAA: Health Insurance Portability and Accountability Act
NVB: nonvalues-based
PrepDM: Preparation for Decision-Making
PS: problem-solving measures
PSDM: problem-solving and decision-making measures
REDCap: Research Electronic Data Capture
SDM: shared decision-making
SRIS: Self-Reflection and Insight Scale
VB: values-based
VECTORS: Values Elicitation and Clarification of Treatment Options Rating Scale
VOICE: Values and Outcomes to Improve Cancer Experiences
