# Supplementary material for: Advancing Knowledge of Values-Clarification Processes During Complex Decision-Making Among Older Adults With Advanced Cancer: Protocol for a Pilot Randomized Trial Using Simulated Patient-Clinician Encounters
Source: JMIR Res Protoc. 2025 Oct 27;14:e80531. doi: 10.2196/80531 (PMC12603586; doi:10.2196/80531)
Supplement: Multimedia Appendix 1 [file resprot_v14i1e80531_app1.pdf]

Values and Outcomes to Improve Cancer Experiences (VOICE): a digital values-clarification tool
Includes Best-Worst Scaling (BWS) survey and tailored summary report

When you think about making a treatment decision, what is most important to you? Choose one outcome that is most important and one outcome that is least important to you.

Most Important		Least Important
○	Doing activities that are meaningful to me	○
○	Managing my medical expenses	○
○	Maintaining my independence	○
○	Minimizing side effects	○

When you think about making a treatment decision, what is most important to you? Choose one outcome that is most important and one outcome that is least important to you.

Most Important		Least Important
○	Minimizing side effects	○
○	Reducing my time spent receiving care	○
○	Accessing resources for understanding my treatment plans	○
○	Living longer	○

(a) Example of BWS survey, displaying all seven treatment values

What's Most Important to You

Based on the results of your survey, **maintaining independence** and **doing activities that are meaningful** are most important to you right now.

Before making a treatment decision, remember to speak up about the following **3 things** and let your clinician know you want to:

1. **Discuss what you value most** when making a treatment decision. Say, maintaining independence is important to me because I want to be able to...

2. **Ask questions.** Based on your responses to this survey, we encourage you to ask the following questions to learn how "what you value most" is impacted by different treatment options.

   How will each treatment option affect my ability to maintain my independence and limit the assistance I need from others?

   What can I do to improve the likelihood that I will maintain my independence and reduce the need for assistance, and will this vary by treatment options?

   Are there options to modify my treatment plan to improve the likelihood that I will maintain my independence and reduce the need for assistance?

   How will each treatment option affect my ability to participate in the activities that are important to me, and what changes might I need to make to keep doing them?

3. **Understand.** In your own words, tell the clinician your understanding of how the things you value most will be impacted by each treatment option. We believe this will ensure that you and your clinician are on the same page regarding your treatment plans.

The content within this decision support tool was developed by patients, caregivers, and clinicians.

Bring this results page with you to your upcoming visit to help you remember to discuss what is most important to you, ask questions, and ensure you understand how your values are impacted by different treatment options.

(b) Example of tailored summary report from VOICE
